# The setting up and running of a children's activity group and learning centre for children with haematological and oncology conditions

**DOI:** 10.1002/cnr2.1511

**Published:** 2021-08-25

**Authors:** Rhea Daruvala, Valli Narasimha, Sandhya Sharad, Manjari Bhatnagar, Prem Kumar, Sunil Bhat

**Affiliations:** ^1^ Department of Pediatric Hematology, Oncology and Bone Marrow Transplants Mazumdar Shaw Cancer Center Bangalore Karnataka India; ^2^ Samiksha Foundation Bangalore India

**Keywords:** activity group, childhood cancer, education, psychological well being

## Abstract

**Background:**

Children diagnosed with haematological and oncology conditions spend long periods of time undergoing treatments in hospital. Treatments are intensive and may include combinations of chemotherapy, radiation, surgery and bone marrow transplants. This often means that they have prolonged hospital stays away from family, friends and familiar environments.

**Aim:**

We aimed at starting an activity group and learning centre based in the hospital setting for children undergoing treatment for haematological and oncology conditions.

**Methods:**

The activity group and learning centre was set up in a tertiary care hospital under the department of Paediatric Haematology, Oncology and Bone Marrow Transplantation with the support of a local NGO called ‘Samiksha Foundation’ in Bangalore, India. Children who participated in the programme engaged in learning through participation in the activity groups which engaged in academic and non‐academic activities. The activity group and learning centre was piloted in April and May of 2019. During the pilot sessions, 156 children participated in the group. Children of all ages were welcome to attend and were given activities based on their age and learning levels. Until March 2020, the group has seen over 600 children in attendance.

**Results:**

This methods report examines various aspects of the activity group such as setting up of the activity groups, how they are run, the activities conducted in the groups and the effects the group has had on children and their families as reported by them while undergoing treatment.

**Conclusion:**

The overall response to the activity groups was positive and widely accepted among our patient community. The intervention proved to be effective, easy to implement and relatively inexpensive. We hope that by sharing data from our centre, more paediatric units may be able to implement such groups for children.

## 1 INTRODUCTION

Childhood cancers are known to be one of the leading causes of death worldwide and affect approximately 300 000 children ages 0–19 years each year. Although these numbers may seem alarming, childhood cancers have high cure rates when treated in an effective and timely manner. In the developed world, 80% of children with cancer are cured (WHO, 2018).

Treatments for childhood cancers are long drawn and often include a combination of chemotherapy, radiotherapy, surgery and sometimes bone marrow transplants. This means that children spend long durations of time in hospital or in close proximity to the hospital while being treated for the illness. And often children and their families are often away from home. It has been found that children with chronic illnesses usually have educational needs that are neglected.^1^ Several studies have shown that non pharmacological interventions, play based activity, music based activity etc are effective for children undergoing hospitalisations in various ways such as anxiety management, coping, improved well‐being.^2^, ^3^, ^4^, ^5^, ^6^, ^7^

Hospitalised children often find themselves in new and unfamiliar environments coupled with the anxiety of medical procedures and side effects of treatment. This could be stress inducing for children as well as their families. Play therefore often acts as a ‘safe space’ or a time out from the anxiety inducing hospital environment. Play in hospital settings has been found to have several benefits including reducing stress and anxiety, helps in maintaining the child's self‐esteem and confidence, contributes in the development of new creative solutions to problems they face, encourages participation of families, helps build a community of support in the hospital and importantly provides amusement and joy to children.^6^, ^7^, ^8^, ^9^, ^10^ This methods report examines various aspects of the activity group such as setting up of the activity groups, how they are run, the activities conducted in the groups and the feedback received from families and caregivers undergoing treatment for haematological and oncology conditions.

## 2 METHODS

The main aim of setting up the children's activity group and learning centre was to ensure that children are engaged academically, emotionally and creatively during the course of their treatment. Since treatment for paediatric cancers and haematological illnesses are often long drawn leading to children missing out on long periods of school, socialisation and activity. Unless engaged, getting back to normal life and a school routine can often be difficult. Oftentimes children are left behind in the same grade while their peers have been promoted thus affecting self‐esteem and confidence levels which are often already low due to the effects of treatment on a child's physical and emotional health.

The activity group and learning centre therefore aimed at fulfilling this gap in learning while children undergo treatment. We (the Department of Pediatric Hematology, Oncology and Bone Marrow Transplant) partnered with a local not‐for profit organisation that helped in providing teachers and volunteers to aid in the learning process. The teachers were qualified government school teachers while volunteers were working professionals and university students. Volunteers were from several different educational backgrounds, geographical locations, and professions. This also allowed children to interact and understand different goals they could have in terms of their futures and careers along with having role models to look up to. Having a set of well qualified teachers and volunteers was necessary since the children who were part of the activity group and learning centre ranged from 3 to 18 years of age and their educational and learning needs required varied expertise. Children from similar age groups, and learning levels were grouped together. Language barriers were also a challenge as the children attending the groups were from various parts of the country and often did not relate to English as their primary language of communication. Volunteers therefore were fluent in various Indian languages such as Hindi, Tamil, Kannada, Telugu and Malayalam and Bengali. The teachers helped in planning the academic activities, which the volunteers were then able to deliver.

The team at the hospital which consisted of the psycho‐oncologist, nurses and doctors helped to identify and encourage children who could attend and benefit from the programme. The team also helped in managing the logistics of the learning centre. Overall, the activity group and learning centre was a coordinated effort by several members, which allowed for it to run smoothly.

Unlike conventional school, academic topics were delivered and learned concept wise by the children as this saved time and allowed for children to complete their curriculum. This helped children understand and learn many important concepts that would usually be covered through their school year rather than the conventional way of learning ‘chapter wise’ from pre‐designed textbooks. The subject matter was based on the national curriculum itself. Children were given worksheets and activities so as to practice and retain the information they learnt. The primary reason for following this method was to keep in mind that children undergoing treatment would not be able to engage in learning as they normally would at school especially with regard to the time they set aside for school and academics. The main objective of the group was to ensure children are on track with formal schooling and are able to cope when they return to their schools. The group was not a substitute for regular school.

### 2.1 Setting

The children's activity group and learning centre was implemented in a tertiary care, private hospital in Bangalore, India under the Department of Pediatric Hematology, Oncology and Bone Marrow Transplants. Majority of children who received treatment at the hospital were undergoing treatment for various haematological and oncology conditions. Some children were also post treatment and regularly following up with the treating team. Being a relatively large centre, children and their families travelled from across the country and neighbouring countries such as Bangladesh, Pakistan, The United Arab Emirates and so on to undergo treatments. The children's activity group and learning centre ran in the playroom of the hospital. This dedicated area was close to the paediatric wards and easily accessible to children and their families. Visiting the play room for activities also allowed children a change of environment from the wards. The play room is colourful, filled with toys and activities and allows children a break from the ward which is usually mundane and associated with drugs, treatment, chemotherapy, injections, and so on.

All children admitted under the department were welcome to attend the group either when they were admitted to the hospital wards or when they were at home and following up in the clinic.

### 2.2 Planning the activity group

The activity group and learning centre was planned collaboratively by the local paediatric haematology, oncology and bone marrow transplant team along with a local not for profit organisation in the education sector. The psychologist on the team coordinated and planned the functioning and running of the group along with a team from the local not for profit organisation. A similar programme run by the same not for profit organisation was also run in another government hospital in the city. The setting and care practices of both hospitals was different and hence a tailored model had to be developed.

Since the children who participated in the group were all patients and were not always admitted to hospital, the team devised a way to keep track of each child's progress so that they could have continuity with regards to their work in the group. A system was therefore devised so that each child's work and progress was recorded. This ensured that even when a child was unable to attend the group for a few sessions, he/she was able to re‐start his learning where he/she left off. Most of the reasons as to why the children missed attending the group was related to their health and therefore a very strict attendance policy was difficult to implement.

### 2.3 Setting up the pilot phase of the activity group and learning centre

During the pilot phase, we decided to engage the children admitted to the general ward of the hospital and engage with them twice a week. Each session was 2–2.5 h long. Children were encouraged to complete the entire session however, on some occasions children had to leave early, start late or take a break in between for various reasons that were treatment related and could not be postponed. During the session children completed some academic work as well as some activity based learning. If the child finished their scheduled portion for the day they were allowed to engage in any activity they enjoy such as playing chess, doing a puzzle, drawing, painting etc. The primary caregiver, most often the mother was also invited to be part of the session. Mothers too engaged in art and craft and most often requested this on their own. They were also able to help and supervise their child's activity.

The pilot phase was run successfully for a 2 months period. Children and their parents attended these groups regularly and provided feedback which was suggestive that the groups were helpful and positively appreciated by families. Data from the pilot phase is captured in the table below which shows the number of children that attended the group and the grade of the children.

### 2.4 Setting up the permanent activity group and learning centre

Since the pilot phase of the activity group and learning centre was successful with a positive demand for the group to continue, we decided to introduce the group to all paediatric haematology, oncology wards in the hospital with the exception of the bone marrow transplant unit for reasons of infection control. The group was conducted thrice a week. This helped us to ensure every child received adequate attention during the group and the number of children per group was not too large. Children who were admitted were always encouraged to be a part of the group. They were escorted to the play room of the hospital where they would engage in the group. Children who were sick or in ICU were exempted. The group activities were conducted in a similar manner as in the pilot phase with teachers and volunteers covering a concept each day. These concepts were covered through teaching, activity based learning and worksheets. With the inclusion of a third day, we were able to cover a larger part of the academic portion for the children. We also introduced some ‘fun days’ every quarter wherein resources were hired to entertain the children. These included puppet shows, music sessions, drama and theatre, singing workshops, and so on.

## 3 RESULTS AND DISCUSSION

The hospital‐based children's activity group and learning centre we aimed to establish and run was quite unique in its concept. The main aim was to provide children being treated for childhood cancers to be able to continue their education as well as engage in play and activity while being in hospital or away from their homes. The activity group and learning centre provided this opportunity as children could engage in learning and education so that they could continue formal schooling when they completed treatment. The group was not a substitute for regular school however helped ensure that they are on track with their education and do not have to repeat a year when they return to school. Majority of children continued to be enrolled in their schools while undergoing treatment.

During the pilot phase which was conducted in April and May of 2019 we saw a total of 156 children attend these sessions. These sessions were conducted across 8 weeks, twice each week. During the pilot phase, we found that the majority of children who attended the group were from younger age groups, majority of children (*n* = 76) being in the 4–8 years age range. This was interesting as children in this age group are often learning basic concepts such as numbers, the alphabet, basic spelling, and so on which contribute significantly to their academic foundation. The table below shows the number of children the activity group and learning centre had in attendance during the year along with the age range of the attendees (Table 1).

**TABLE 1:** The number of children as per their age and academic grade who attended pilot phase

Age group	Number of children who attended the group
Under 3 years of age	31
Kindergarten (approx. 4–5 years old)	52
Grades 1 and 2 (approx. 5–7 years old)	24
Grades 3 and 4 (approx. 7–9 years old)	16
Grades 5 and 6 (approx. 9–11 years old)	10
Grades 7 and 8 (approx. 11–13 years old)	13
Grades 9 and 10 (approx. 13–15 years old)	8
Grades 11 and 12 (approx. 15–18 years old)	3
TOTAL	156

We also found that 31 children aged 2–3 years participated in the group. These younger children were always accompanied by their parents or caregiver and engaged in play‐based activity which also helped in learning. They were provided with play‐based activities which helped in developing their motor skills, auditory and visual stimuli to help keep them engaged as well as learn and of course play as they enjoyed.

Older children in the group were fewer in numbers and preferred to learn on their own. They were given tasks for the day which they were encouraged to complete as well as worksheets for further practice. They were aided by teachers and volunteers in subject areas they individually found particularly challenging such as math and science.

Since the pilot phase was successful and feedback from several parents and children was positive, the team decided to launch the activity group and learning centre on a regular basis. Some of the feedback received from parents and caregivers of children is as follows.

the origami paper craft activities were fun and engaging. Our children learnt about animals in a creative way. We all had fun (mother of patient S, 5 year old)

Enjoyed the activity and learned new topics. It was a big distraction for my child and me. Even though he was not well, he was happy and did the activity (Father of patient T, 7 year old)

the activity was very innovative. My baby learned about windmills and renewable sources of energy while doing craft. Thank you and hope you continue this (Mother of patient V, 11 year old)

The regular group was introduced in June of 2019. The group runs three times a week, in two batches each to cater to the larger number of children. The group was scheduled for Wednesday, Friday and Saturday every week. Once this group was introduced, we saw several children attend this group regularly. Children who were not currently admitted to hospital but lived close by began coming to hospital exclusively for the group and wanting to engage in school work. We documented the attendance data for children from June 2019 to mid‐March 2020 as shown in Table 2 below.

**TABLE 2:** The number of children who attended the group from June 2019 to March 2020

Age group	Number of children who attended the group
Under 3 years of age	149
Kindergarten (approx. 4–5 years old)	85
Grades 1 and 2 (approx. 5–7 years old)	88
Grades 3 and 4 (approx. 7–9 years old)	62
Grades 5 and 6 (approx. 9–11 years old)	68
Grades 7 and 8 (approx. 11–13 years old)	47
Grades 9 and 10 (approx. 13–15 years old)	24
Grades 11 and 12 (approx. 15–18 years old)	11
TOTAL	534

The younger age groups again seemed to be the most common in the activity groups. However, it was observed that older children attended the groups more regularly and frequently. They also expressed a keen interest in the group's activities. With the introduction of online classes after the COVID‐19 pandemic several children were able to re‐join school remotely and were promoted to the next grade as they were able to successfully complete their final examinations. This could be attributed to the regular learning and coursework they were able to complete through means of the activity group. Parents too were more involved in the group ensuring that their children completed the worksheets given to them, making sure they brought their books to the group and asked for assistance.

Besides the children and parents who participated in the group, volunteers too found the experience fulfilling.

Volunteering gave me an opportunity to be a part of the teams mission of educating and caring for children with cancer. This is one of the best eye opening experiences for me where I saw the determination and liveliness of kids even after suffering from such a life threatening disease.

Volunteering gave me an opportunity to improve my skills and apply my theoretical understanding in real life and also gain a perspective on how it is to work with kids and to be able to assist and help them out with various activities effectively. Initially it was all new to me but after interning for a few weeks I was able to get the hang of it and learnt how to effectively communicate with the kids.

## 4 CONCLUSION

The children's activity group and learning centre which was established in June 2019 for children being treated for childhood cancers was a learning experience for every stakeholder. The group has proven to be an important part of the children's treatment as they most often look forward to the fun and learning that takes place. Children and families also made friendships during the course of the group. The most significant advantage and learning from the group for the team was that this was a relatively easy‐to‐implement group and also a cost effective measure for children undergoing long treatments for various illnesses. Implementing a group such as this allows children the opportunity to continue their education despite being away from physical school. The limitations of the group however must not be ignored. The group required significant man power to cater to the varied learning needs, language requirements and age groups of the children. Space constraints also proved to be challenging once the group became popular among the patient community. Overall, the activity and learning centre is easily replicable to other centres that have similar populations of children undergoing long treatments.

## CONFLICT OF INTEREST

The authors declare there is no conflict of interest. No external funding or monetary benefits were obtained with regards to the publication of this report.

## AUTHOR CONTRIBUTIONS

Conceptualization, R.D, V.N, P.K. and S.B.; Methodology, R.D., S.B.; Formal Analysis, R.D., V.N.,P.K., M.B., and S.S.; Resources, R.D., V.N.,M.B.,S.S., and S.B.; Writing ‐Original Draft, R.D., and V.N.; Writing ‐ Review & Editing, R.D.,V.N., And S.B.; Supervision, S.B.

## ETHICS STATEMENT

The current report has not used any patient data and no tools, instruments or tests were performed on any participants. All data provided is retrospective in nature and did not require clearance from an ethics committee. Ethical clearance was obtained from the Ethics Committee at the hospital.

## Data Availability

The data that support the findings of this study are available from the corresponding author upon reasonable request. All data presented and analysed is available within the manuscript.
